# Binding of a Novel 11β-Hydroxysteroid to the Apelin Receptor (APLNR) and Its Possible Role in Regulating Skeletal Muscle Health in Mice

**DOI:** 10.3390/ijms27209379

**Published:** 2026-10-21

**Authors:** Marcos González-Pérez, Citlali Gómez-Márquez, Miguel Ortiz-Flores, Marycarmen Godínez-Victoria, Nayelli Nájera, Guillermo Ceballos

**Affiliations:** 1 Laboratorio de Investigación Integral Cardiometabólica, Escuela Superior de Medicina, Instituto Politécnico Nacional, Mexico City 11340, Mexico; 2 Sección de Posgrado, Escuela Superior de Medicina, Instituto Politécnico Nacional, Mexico City 11340, Mexico

**Keywords:** apelin receptors, skeletal muscle, 11β-hydroxypregnenolone

## Abstract

Several molecules have been investigated for their potential to improve muscle health in models of muscle damage simulating sarcopenia, injury, or aging. Some small molecules induced beneficial effects on skeletal muscle by stimulating the apelin receptor (APLNR). This receptor has emerged as a promising pharmacological target for modulating muscle homeostasis. Using an in silico approach, we recently identified 11β-hydroxypregnenolone (11β-OHP5) as a potential skeletal muscle (SKM) modulator. We hypothesized that 11β-OHP5 interacts with and activates APLNR. This study employed several research approaches: (1) in silico molecular docking and molecular dynamics to explore the feasibility of 11β-OHP5 interacting with APLNR; (2) in vitro activation analysis to APLNR; (3) in vitro analysis C2C12 myoblast differentiation; (4) analysis of the molecule’s presence in mouse skeletal muscle and its differential presence between young and older mice. The results show that 11β-OHP5, acting through APLNR, positively affects myoblast differentiation and may participate in the exercise-induced benefits in aging mice. We show that 11β-OHP5 interacts with APLNR, induces myogenic responses, is found in SKM; its concentration decreases with age, and exercise restores it. More work is necessary to understand the physiology of this possible endogenous molecule.

## 1. Introduction

Recently, several molecules have been investigated for their potential to improve muscle health, with (-)-epicatechin (EC), a flavanol extensively characterized by our group, standing out for its beneficial effects on skeletal muscle. In murine models of muscle damage simulating sarcopenia, injury, or aging [1,2,3,4], EC accelerates recovery, induces muscle fiber hypertrophy, and increases the expression of key myogenic factors such as Myoblast determination protein 1 (MyoD) and myogenin [5]. It also downregulates catabolic signaling pathways associated with atrophy, including Forkhead box 01 (FOXO1a), myostatin, and Muscle ring-finger protein-1 (MuRF1) [6,7]. In C2C12 cells, EC promotes differentiation into myotubes by activating key regulators of myogenesis [8].

Within this framework, our group identified 11β-hydroxypregnenolone (11β-OHP5), a molecule with no previously reported biological activity and a structure closely resembling EC. Remarkably, this compound increased the expression of mitochondrial complexes II and IV, suggesting mitochondrial effects, with greater potency than EC and achieving maximal effects at nanomolar concentrations [9]. To date, to the best of our knowledge, this remains the only study documenting its potential actions, leaving its role in muscle biology largely unexplored.

Recently, we have shown that many EC-induced effects are mediated through specific stimulation of the apelin receptor (APLNR) [10], a G protein–coupled receptor widely expressed in skeletal muscle tissues [11]. This receptor has emerged as a promising pharmacological target for modulating muscle homeostasis, as its activation promotes mitochondrial content and muscle fiber formation [12,13].

Based on the structural and functional similarities between EC and 11β-OHP5, and the growing interest in identifying compounds that promote muscle differentiation and maintain a healthy muscle phenotype, we hypothesized that 11β-OHP5 interacts with and activates APLNR, thereby inducing myogenesis, and that it can modulate SKM function. This study employed several research approaches: (1) in silico molecular docking and molecular dynamics to explore the feasibility of 11β-OHP5 interaction with APLNR; (2) in vitro (CHO-K1 cells transfected and stably expressing the receptor) functional activation analysis; (3) in vitro analysis of 11β-OHP5 effects on C2C12 myoblast differentiation; (4) analysis of the presence of 11β-OHP5 in mice skeletal muscle and its differential presence between young and older mice and the differential concentration in skeletal muscle of 24 month old mice under sedentary or exercising conditions.

The results reported here showed, for the first time, the positive effects of 11β-OHP5, exerted through APLNR, on myoblast differentiation and its possible participation in the exercise-induced positive effects in aging mice.

## 2. Results

First, to characterize the possible interaction of 11β-OHP5 with the activated apelin receptor (aAPLNR) form, we performed molecular docking simulations. We established a reliable docking protocol by re-docking the native co-crystallized ligand (cmpd644) into the reference structure (PDB: 7W0L), confirming it reproduced the experimental pose with an RMSD below 2.0 Å. Using this validated protocol, we compared the binding modes of 11β-OHP5, its precursor (P5), and the reference agonist within the same binding cavity. The analysis revealed five interactions for 11β-OHP5 with residues Y35, W85, I109, F110, and Y264. Notably, 11β-OHP5 shares the F110 and Y264 interactions with cmpd644 and the W85 and Y264 interactions with P5 (the only two interactions observed for this molecule). This consensus and energetically favorable pose for 11β-OHP5, suggestive of an agonistic mechanism of action, was selected as the starting point for subsequent molecular dynamics analyses (Figure 1).

While docking provided static interaction profiles, we assessed the stability of the selected binding pose through all-atom molecular dynamics (MD) simulations. The aAPLNR:11β-OHP5 complex was simulated for 1 µs. For comparative purposes, the ligand was docked into the inactive receptor conformation using the same protocol and grid parameters described above and simulated under identical conditions. In both systems, the ligand remained within the orthosteric pocket throughout the trajectory (ligand RMSD < 0.7 Å), supporting the stability of the modeled poses.

Interaction energies were estimated by MM/GBSA over 101 evenly spaced frames (10 ns intervals) drawn from the production trajectories. The calculated values were more favorable for the active conformation (−23.91 ± 0.56 kcal/mol) than for the inactive one (−12.61 ± 0.87 kcal/mol), where the reported uncertainties are standard errors of the mean obtained by block averaging, which accounts for the autocorrelation of the energy time series (autocorrelation times ≤ 10 ns and 20 ns, corresponding to approximately 38 and 26 effectively independent samples, respectively).

With the interaction-entropy approximation, the estimated binding free energies were −8.29 kcal/mol for the active conformation and +3.25 kcal/mol for the inactive one, with estimator dispersions of 3.64 and 4.63 kcal/mol. Although the interaction-entropy term converges slowly and these absolute values therefore carry substantial uncertainty, the qualitative distinction was consistent across the analyzed frames: binding remained favorable in the active conformation and became unfavorable in the inactive one. We note that these are end-point estimates derived from a single trajectory per system. Accordingly, we use them for qualitative comparison between conformations, not as estimates of absolute binding affinity; establishing state selectivity would also require accounting for the free-energy cost of the receptor conformational transition.

Per-residue energy decomposition indicated that R168 contributed favorably in both conformations, suggesting a conserved anchoring interaction. In the active conformation, F291 and P292 provided the additional major contributions, whereas in the inactive conformation the second principal contributor was W85, consistent with pocket rearrangement between the two states. It has to be noted that these computational results are exploratory and performed only in the binding pocket of APLN. Table 1 summarizes the individual energy contributions for all key residues.

Before analyzing conformational features, we verified that the transmembrane bundle retained its architecture over the simulated timescale. Per-helix backbone RMSD remained low for all seven transmembrane helices in both systems (Table S1), and the solvent-accessible surface area of hydrophobic transmembrane residues showed no progressive increase, with mean values of 164.3 ± 5.0 nm^2^ (aAPLNR) and 180.6 ± 3.9 nm^2^ (iAPLNR) and a slight net decrease across the trajectories (Figure S1).

Next, to characterize the structural features distinguishing the two receptor states and their relationship to effector-binding site geometry, we analyzed key conformational parameters. Comparison of the experimentally determined active and inactive APLNR structures showed the canonical class A activation pattern: in the active state, the intracellular end of TM6 is displaced outward, away from the receptor core, relative to the inactive state. ICL3 adopts an extended conformation directed away from the receptor core, in contrast to its folded arrangement in the inactive state. Together, these rearrangements enlarge the intracellular cavity that accommodates effector proteins. The inward positioning of TM7 toward the receptor core further shapes this cavity (Figure 2).

To examine whether these distinguishing features were maintained in the ligand-bound systems, we compared the majority clusters derived from the MD trajectories of the two states. The characteristic differences were preserved in the presence of 11β-OHP5: relative to the inactive complex, the active complex retained (i) an outward position of the intracellular end of TM6, (ii) an extended ICL3 conformation, and (iii) an inward position of TM7, accompanied by a repositioning of H8 toward the receptor core (Figure 3).

We quantified these differences as follows. The flexion angle of the intracellular segment of TM6, measured from the alpha carbons of L247, C260, and I66, with C260 as the vertex, averaged 42.38 ± 1.72° in the inactive complex and 69.23 ± 1.96° in the active complex. The distance between the hydroxyl groups of Y221 (TM5) and Y309 (TM7) averaged 17.888 ± 0.79 Å and 8.409 ± 1.14 Å in the inactive and active complexes, respectively (Figure 4). These measurements quantify the structural separation between the two ligand-bound states over the simulated timescale.

Finally, as an exploratory structural analysis, we modeled the docking of G_i_ protein and β-arrestin onto the majority clusters of both states to examine the geometric compatibility of the intracellular cavity with each effector. R127 occupied a comparable position in both complexes. In the active-state model, the outward position of TM6 and the extended ICL3 conformation relieved the steric overlaps that obstructed effector placement in the inactive-state model, while the reorientation of H8 introduced minor residual overlaps (Figure 5). Within the limits of this static analysis, the active-state conformation presented a geometrically accessible surface for both effectors, with no evidence of preferential compatibility with either one. We emphasize that these are geometric observations derived from representative structures and do not constitute evidence regarding effector recruitment or signaling bias, which would require experimental validation.

### 2.1. Receptor-Dependent Signaling Induced by 11β-OHP5 Interaction with APLNR Through the β-Arrestin Recruitment Assay

We developed a 11β-OHP5 concentration/response curve in CHO-K1 cells transfected to stably express the APLNR receptor, using Apelin-13 as a positive control. The signaling patterns of apelin and 11β-OHP5 show similar characteristics, with Apelin ED_50_ = 4.21 e^−15^ M and 11β-OHP5 ED_50_ = 1.68 e^−15^ M. Interestingly, ML221 [1 µM], an antagonist of APLNR, shifted the concentration–response curve to the right, suggesting a competitive inhibition of 11β-OHP5 stimuli with EC_50_= 5.83 e^−8^ M (Figure 6A).

On the other hand, since the in silico analysis suggested similar activation of GPCR and β-arrestin pathways when 11β-OHP5 interacts with APLNR, we decided to explore the activation of GPCR pathways in a concentration/response, receptor-dependent signaling-induced by 11β-OHP5 curve in CHO-K1 cells expressing the human Apelin receptor (Gi) to measure GPCR activity via decreased levels of intracellular cAMP.

In this analysis, Forskolin is used to activate cells, inducing an increase in cAMP levels (increase in chemiluminescence), our results showed a decrease in luminescence, suggesting a concentration-dependent decrease in cAMP and specific binding, since the presence of ML221 [1 µM] blocks the cAMP decrease (Figure 6B). To validate these in silico and activation results, we investigated the effects of 11β-OHP5 on a biological model: the differentiation of mouse C2C12 myoblasts. The results suggested increased differentiation, as shown in Figure 7.

RT-qPCR revealed that after 8 days of stimulation under forced differentiation conditions, 11β-OHP5 administration increased expression of the myogenic markers myosin and dystrophin (Figure 8A,B). This effect followed a biphasic response curve, reaching maximum efficacy at 0.1 nM for both markers. We then used this concentration in all functional experiments.

To confirm that the pro-differentiation effect of 11β-OHP5 was mediated through APLNR activation, myoblasts were pretreated with ML221, a full receptor antagonist [1 µM]. Blockade of APLNR with ML221 abolished the 11β-OHP5-induced increase in myosin and dystrophin expression (Figure 9A,B), reducing these markers to levels comparable to the untreated control groups. This result confirms that signaling through APLNR is essential for 11β-OHP5 myogenic activity.

Having established this dependency, we next sought to delineate the specific contribution of the β-arrestin pathway to the myogenic response using Barbadin, a selective inhibitor that interferes with β-arrestin binding to the receptor. Quantification revealed that barbadin treatment significantly attenuated the effect of 11β-OHP5, reducing myosin and dystrophin expression by approximately 45% and 25%, respectively, compared with the agonist-alone condition. These findings suggest that the APLNR-mediated pro-differentiation activity of 11β-OHP5 integrates two signaling pathways: one dependent on β-arrestin and another independent of it (likely mediated by G proteins) (Figure 9A,B).

#### Animal Model

The previous results showed specific receptor stimuli and myogenic stimuli induced by of 11β-OHP5; however, those results do not allow us to propose any in vivo effect or presence, so in consequence to analyze the presence of 11β-OHP5 in vivo we use several approaches: (1) show presence of the steroid in suprarenal glands, since this tissue is the main source of pregnenolone and we believe that 11β- hydroxylation can or must be done in this tissue; (2) Since we showed a myogenic stimuli in mice myoblasts we analyzed steroid presence in extracts obtained from skeletal muscle of young (3 months old) and, 24 months old mice, sedentary or under a weekdays exercise regimen (starting with 3 months old animals and following them until they reached 24 months of age) to analyze changes related to ageing and ageing under exercise.

The results were obtained from suprarenal gland extracts using HPLC separation coupled to mass analysis, with pregnenolone and commercially available 11β-OHP5 as internal standards, demonstrating the presence of 11β-OHP5 in suprarenal tissue.

We validated the range limits according to ICH Q2(R2) guideline; the detection limit (DL) and quantitation limit (QL) were determined based on the standard deviation of the linear response and the slope of a calibration curve according to the formulas: DL = 3.3σ/S and QL = 10σ/S, where σ equals the standard deviation of residuals of the regression line and S equals the slope of the calibration curve. The standard curve was built with seven concentrations ranging from 5 to 25 µg/mL, each in triplicate, and adjusted by ordinary least squares (r^2^ = 0.9927). The QL was verified by an independent analysis of 6 replicates, yielding an RCV of 1.19%. Thus, DL = 2.51 µg/mL and QL = 7.62 µg/mL were obtained.

The analysis of the extracts obtained from gastrocnemius muscles is shown in Figure 10A, the comparative analysis of the concentration of 11β-OHP5 suggest that there is a decrease in this molecule in the muscle that is dependent in the age of the analyzed mice since the concentration found in young mice is significant higher than in 24 months old mice, remarkably the exercise regimen kept diminish the decrease in 11β-OHP5, even when the concentration is still different as compared with young animals these results clearly demonstrate that exercise is a good if not the best way to diminish or recover the concentration of this steroid.

To assess the possible contribution of 11β-OHP5 and/or exercise in the 24-month-old sedentary vs. exercised groups, we analyze changes in dystrophin/mg of protein in the skeletal muscles of these groups. Figure 10B shows a significant exercise-induced change in dystrophin concentration, demonstrating the relevance and influence of exercise and/or 11β-OHP5 in maintaining mobility capacity in aged mice.

## 3. Discussion

The main results of this study demonstrate the presence of 11β-OHP5 in animal skeletal muscle; its theoretical and functional activation of APLNR; a decrease in its concentration during aging; and a possible recovery of its presence induced by exercise in elderly mice.

Our study demonstrates that the molecule 11β-OHP5 may act as an agonist of the APLNR receptor, stabilizing its active conformation to integrate activation of both G protein and β-arrestin pathways, culminating in potentiation of myogenic differentiation.

These results not only identified the structural determinants of binding and activation but also revealed a previously undescribed axis linking a steroid metabolite to myogenesis regulation through a specific GPCR.

Molecular docking analysis revealed that 11β-OHP5 shares key interactions with the reference agonists (residues F110, Y264) and with its precursor, pregnenolone (P5) (residues W85, Y264). In dynamic characterization via 1 µs MD simulations, we quantified the notable preference of 11β-OHP5 for the active conformation (aAPLNR) over the inactive one (iAPLNR), as evidenced by a significantly more favorable binding free energy (ΔG) (−23.91 vs. −12.61 kcal/mol). Energy decomposition per residue identified R168, F291, and, notably, P292 as the main contributors to active-complex stability. The interaction with R168, a residue reported as a determinant for G protein bias [11], suggests a structural mechanism that predisposes the activation of this pathway. Conversely, the stabilization of the inactive complex, albeit weaker, involved I109, a residue previously associated with β-arrestin bias in APLNR [11], indicating that 11β-OHP5 can stabilize multiple states, albeit with a clear preference for the active form.

The structural findings align with the contemporary model positing that GPCRs exist in a dynamic conformational landscape, where biased ligands stabilize subsets of states [14]. The outward movement of TM6 and the extension of ICL3 induced by 11β-OHP5 represent canonical features of class A GPCR activation, consistent with structural studies on APLNR and other receptors [15,16,17,18]. However, the marked energetic preference for the active state (−23.91 kcal/mol) surpasses the typical stabilization reported for natural agonists, suggesting a distinctive pharmacological profile.

The observation of a concurrent inward movement of TM7 and rearrangement of Helix 8 (H8), structural changes frequently associated with β-arrestin-biased signaling [14,15], coupled with the significant energetic contribution from residue R168 (linked to G-protein bias), provides a mechanistic explanation for the balanced (not biased) signaling profile we observed functionally. This contrasts with clearly biased ligands, such as some apelin analogs, which report a more extreme conformational preference towards one pathway or the other [10,11,15]. Our data suggest that 11β-OHP5 occupies a unique pharmacological niche, stabilizing a “complete” yet non-extreme active state that remains accessible to both effectors (G-proteins and β-arrestin).

Functional validation supports the structural prediction that the 11β-OHP5-stabilized active state enables coupling to both effectors, first in the activation analysis, where we show that 11β-OHP5 stimulates both pathways (11β-OHP5- and G-protein-related), a result reaffirmed in the C2C12 myoblast differentiation model. Interestingly, treatment with 11β-OHP5 significantly potentiated myogenesis, exhibiting a biphasic response curve as previously reported by Dugar [9]. This effect was APLNR-dependent, as shown by its blockade with the antagonist ML221. We also showed, using the selective β-arrestin inhibitor, barbadin, the relative contribution of each pathway in this C2C12 model: while the G protein pathway was predominant (particularly for the expression of dystrophin, a terminal muscle maturation marker), the β-arrestin pathway contributed (~45%) to myosin expression. These results corroborate the conformational model in which 11β-OHP5 acts as an agonist of both pathways; it can activate both APLNR signaling branches to orchestrate an optimal myogenic differentiation program [12,19].

A critical finding underscoring the specificity of 11β-OHP5 was its divergence from its precursor, P5. This positions 11β-hydroxylation not merely as a modifier of affinity but as an essential structural determinant of receptor specificity.

These results allow us to propose the interaction and stimulation of APLNR by 11β-OHP5 as a model for inducing myogenic responses; however, these results do not show its presence or potential use in vivo. To fill this gap, we decided to explore the presence of 11β-OHP5 in the skeletal muscle of young and old mice (24 months old) with two objectives: (1) to demonstrate 11β-OHP5 presence and (2) to find any changes due to the aging process. Finally, we explored changes in 11β-OHP5 concentration induced by the most effective therapy for sarcopenia, exercise. Our results showed the presence of 11β-OHP5 in vivo, suggesting that this molecule may be a natural agonist of APLNR. Interestingly, its concentration decreases with aging, and it can be improved with exercise. The difference in dystrophin expression between sedentary and exercised animals suggests that 11β-OHP5 may help maintain healthy muscle in the elderly, showing that nothing is better than exercise while highlighting the probable influence of this phenomenon.

The results reported in this work support the existence of a steroidal molecule that may be involved in skeletal muscle physiology, and its effects via interaction with APLNR suggest it may circumvent the adverse effects characteristic of steroid hormones acting through canonical receptors on skeletal muscle [20].

On the other hand, identifying other potential target receptors for 11β-OHP5 remains necessary, given that its precursor P5 and the structurally similar molecule EC can interact with the pregnane X receptor (PXR) [8].

A limitation of this study is the static nature of the effector-complex modeling, which does not capture the full dynamics of the coupling process, suggesting that more in silico work is needed, including analysis with the receptor in membranes. Furthermore, while the C2C12 model is a standard approach for myogenesis studies, extrapolating these results to primary myocytes or muscle tissue in vivo requires deeper validation. Finally, the relative contribution of specific G protein subtypes (Gi vs. Gq) to the balanced profile warrants further characterization.

Another limitation is the unknown source of 11β-OHP5, including direct enzymatic biosynthetic and degradation pathways and those in the backdoor pathway [21]. More work is necessary to fill these gaps.

## 4. Conclusions

Collectively, this work establishes 11β-OHP5 as a possible key endogenous modulator of APLNR function in muscle. Our study not only advances understanding of the pharmacology of a physiologically relevant endogenous steroid but also shows how integrating atomic-scale simulations with cellular models can decipher the nuances of biased signaling in a functional biological context.

## 5. Materials and Methods

### 5.1. Molecular Docking

The initial ligand structures were obtained as follows: pregnenolone (P5; PubChem CID: 8955) and cmpd644 (PubChem CID: 131676386) were downloaded from PubChem [22] in SDF format. The structure of 11β-OHP5 was manually derived from P5 using ChimeraX v1.6 [23] and subsequently converted to PDB format. All ligands were subjected to geometry optimization at the density functional theory (DFT) level with D3BJ dispersion correction using ORCA v5.0.1 [16]. The optimized structures were then converted to MOL files with Chemcraft v1.9 [17] and to PDB files using ChimeraX. Polar hydrogens and Gasteiger partial charges were assigned to all ligands using AutoDock Tools v1.5.7 [24].

The active conformation of the APLNR receptor (APLNRa; PDB ID: 7W0L) was obtained from the Protein Data Bank. Non peptidic components were eliminated, polar hydrogens were added, and Kollman charges were assigned using AutoDock Tools. A grid box with dimensions of 20 × 20 × 20 Å^3^ was defined around the previously identified binding site for cmpd644. Validation of the docking protocol was performed by 10,000 re-docking assays of cmpd644 using SMINA [25], yielding an RMSD < 2.00 Å relative to its crystallographic pose.

Subsequently, 10,000 independent docking assays were performed for each ligand with APLNRa with an exhaustiveness level set to 25. The most frequent binding poses were identified based on the root-mean-square deviation (RMSD) of ligand coordinates within the binding site. These representative conformations were used for comparative analyses of ligand–receptor interactions and for estimating binding free energies (ΔG) with Discovery Studio BIOVIA 2021 [26].

### 5.2. Molecular Dynamics

The receptor and water topologies were generated using the CHARMM36-Jul2020 force field [27]. The topology and parameters for the ligand 11β-OHP5 were obtained from the CGenFF server (https://cgenff.com/, accessed on 1 May 2023). All molecular dynamics (MD) simulations were performed using GROMACS 2023.2. Four independent systems were prepared:

1. The apelin receptor in its active conformation (APLNRa)
2. The apelin receptor in its inactive conformation (APLNRi), obtained from GPCR database (10.1093/nar/gkaa1080)
3. The APLNRa:11β-OHP5 complex.
4. The APLNRi:11β-OHP5 complex.

Each system was solvated in a dodecahedral box with TIP3P water molecules, maintaining a minimum distance of 1.0 nm between the solute and the box boundaries. To emulate physiological conditions, NaCl was added to a concentration of 150 mM, and the system was neutralized by adding the necessary counterions (Na^+^ or Cl^−^). The energy of each system was minimized using the steepest descent algorithm for 50,000 steps with a maximum force tolerance of 10 kJ/mol. Subsequently, the system was equilibrated in two phases: first, a 100 ps simulation in the canonical (NVT) ensemble to stabilize the temperature at 300 K, followed by a 100 ps simulation in the isothermal-isobaric (NPT) ensemble to stabilize the pressure at 1 bar. During both equilibration phases, positional restraints were applied to the heavy atoms of the solute. Production MD runs were conducted in the NPT ensemble for 1 µs per system, a timeframe deemed sufficient to capture relevant receptor conformational changes. We maintained temperature (300 K) and pressure (1 atm) using the V-rescale thermostat and the Parrinello-Rahman barostat, respectively. Long-range electrostatic interactions were calculated using the Particle Mesh Ewald (PME) algorithm. We integrated the equations of motion using the leap-frog algorithm with a 2 fs time step.

Trajectory analysis included conformational clustering to identify the most representative states; calculation of binding free energies using the MM/GBSA method [28,29,30]; and evaluation of specific distances between key amino acid residues using native GROMACS utilities.

### 5.3. Analysis of 11β-OHP5 Activation Induction of APLNR Through the β-Arrestin Recruitment Assay

β-Arrestin recruitment, as a tool for the analysis of 11β-OHP5 binding to APLNR was evaluated using the PathHunter eXpress GPCR kit reader (Eurofins 793-0250C2, Santa Clara, CA, USA). following the manufacturer’s protocol. CHO-K1 cells stably expressing the receptor tagged at the C-terminus with a ProLink^®^ β-galactosidase fragment and β-arrestin-2 fused to the enzyme acceptor fragment were employed. Ligand-induced receptor activation drives β-arrestin recruitment, functionally reconstituting β-galactosidase activity. Hydrolysis of the chemiluminescent substrate generates a proportional luminescent signal. Serial dilutions of agonists and antagonists were prepared in the cell plating reagent and incubated with the cells for 1 h at 37 °C in a 5% CO_2_ atmosphere. The substrate was then added, followed by a 1 h incubation in the dark. Luminescence was measured using a plate reader. Data represent ≥3 independent experiments performed in technical triplicates and normalized to the maximal agonist (Apelin-13) response (100%) and vehicle control (0%).

### 5.4. Analysis of 11β-OHP5 Activation Induction of APLNR Through the G-Protein Pathway

We also used the cAMP Hunter™ Human Apelin Gi Stable Cell Line Assay (Eurofins 795-0147C2, Santa Clara, CA, USA) to measure GPCR activity via decreased intracellular cAMP levels.

This assay uses chemiluminescence to measure ligand-induced activation by quantifying intracellular cAMP levels upon APLNR activation; decreased intracellular cAMP levels lead to a decreased signal in the cAMP detection.

### 5.5. Myoblast Differentiation

A total of 1 × 10^6^ C2C12 cells (ATCC^®^, Manassas, VA, USA, CRL-1772™) were seeded per well (surface area: 9.026 cm^2^) in 6-well plates using high-glucose DMEM supplemented with 10% FBS and 1% antibiotics and incubated at 37 °C in a humidified atmosphere with 5% CO_2_. After 24 h, stimulation with 11β-OHP5 (Steraloids, Newport, RI, USA #Q4590) was initiated under induced differentiation conditions (100% confluence with FBS reduced to 2%) for eight days, with medium changes every 48 h. Initially, a concentration–response curve was performed with 11β-OHP5 at 0 (control), 0.001, 0.01, 0.1, 1.0, and 10 nM. In a second experiment, six groups were established: (1) vehicle (control); (2) 0.1 nM 11β-OHP5; (3) pre-treatment with 1 µM ML221 (Cayman, Ann Arbor, MI, USA), a full APLNR antagonist, for 20 min followed by 0.1 nM 11β-OHP5; (4) pre-treatment with barbadin [1 mM] (AOBIOUS, Gloucester, MA, USA, cat. #AOB3736), a selective β-arrestin recruitment inhibitor, for 20 min followed by 0.1 nM 11β-OHP5. Additionally, stimulation with the direct precursor of 11β-OHP5, pregnenolone (P5; Merck, Naucalpan de Juárez, México # PHR2564), was evaluated in groups: (5) 0.1 nM P5; and (6) pre-treatment with 1 µM ML221 for 20 min followed by 0.1 nM P5. Three independent replicates were performed for each experiment.

### 5.6. RNA Isolation and cDNA Synthesis

Total RNA was extracted from C2C12 cells according to the supplier’s instructions using the Direct-zolTM Total RNA kit from Zymo Research (Irvine, CA, USA). The concentration and purity were subsequently quantified in a NanoDrop 2000 (Thermo Fisher Scientific, Waltham, MA, USA). cDNA was synthesized using the qPCRBIO cDNA Synthesis Kit (PCR Biosystems Inc., East Hartford, CT, USA). 

### 5.7. Quantitative Real-Time PCR (qPCR)

Real-Time PCR Detection System (QuantStudio 1, Applied Biosystems by Thermo Fisher, Waltham, MA, USA). The relative expression of the selected genes was determined using the SYBR Green reagent (BIO-SyGreen; Bio-Rad, Hercules, CA, USA) on a QuantStudio Real (BIO-SyGreen; Bio-Rad, Hercules, CA, USA). Reaction mixtures (final volume: 10 μL) contained 5 μL of SYBR™ Green master mix, 0.5 μL of forward/reverse primer mix (500 nM each) (see Table 2), 1 μL of template cDNA (100 ng/μL), and 3 μL of molecular biology-grade water. The thermal cycling protocol consisted of an initial denaturation at 95 °C for 120 s, followed by 40 cycles of 95 °C for 5 s and 65 °C for 20 s. We calculated relative gene expression using the 2^−ΔΔCt^ method, normalizing to β-actin as the endogenous control. We analyzed all samples in technical triplicates, and each experiment was performed independently three times.

### 5.8. Animal Model

We used male mice aged 3 months (control) and 24 months (sedentary and under an exercise regimen) (starting with 3-month-old animals and following them until they reached 24 months of age).

In search of a positive control, we used suprarenal glands from 3 animals following the next procedure:

#### 5.8.1. Cytoplasm and Mitochondria Isolation

After dissection of suprarenal glands, mitochondria were isolated by differential centrifugation: tissue was excised and homogenized (100 mg/ml) in solution A (KCl 1 M, EDTA 0.1 M, HEPES 0.5 M at pH = 7.4), then centrifuged for 10 min at 800× *g* at 4 °C. Collect the supernatant and centrifuge for 10 min at 8000× *g* at 4 °C. The supernatant was collected and labeled as cytoplasmatic fraction and the pellet was recovered and resuspended in solution B (sucrose 2 M, EDTA 0.1 M, Tris 0.5 M-H_2_PO_4_ 50 mM at pH = 7.4) and centrifuged 10 min × 10,000 g at 4 °C; the mitochondrial pellet was re-suspended in 10 mL of solution C (sucrose 2 M, EDTA 0.1 M, Tris 0.5 M-H_2_PO_4_ 50 mM, succinate 1 M at pH = 7.4).

#### 5.8.2. Hydroxysteroids Isolation

According to the Bradford method for protein concentration determination, we used 500 µg of cytoplasmic or mitochondrial protein for isolation. To support synthetic possibilities in vitro, we added 0.5 M malate, 1 M succinate, and 100 mM ADP to both fractions, then incubated them at 37 °C for 30 min. Afterward, the enzymatic activity was stopped by adding dichloromethane, and a liquid–liquid separation was performed to isolate molecules soluble in the dichloromethane fraction, which were air-dried and evaporated.

#### 5.8.3. Characterization of Soluble Molecules

Molecules in the air-dried dichloromethane fraction were identified by HPLC-MS/MS. Briefly, the fraction was redissolved in HPLC-grade dichloromethane and injected into a Bruker microTOF-Q instrument (Bruker, Bellerica, MA, USA); the scan range was set from 50 to 3000 *m*/*z*, using positive ion polarity (4500 V), electrospray ionization (ESI), and 150 Vpp in the collision cell RF. The mobile phase A was water (buffered with 0.1% formic acid), and the mobile phase B was 100% acetonitrile at a flow rate of 1.0 mL/min.

To analyze the possible presence of 11β-OHP5 in skeletal muscle and possible changes induced by aging or exercise, we used twenty-one 3-month-old male mice (Balb/c). Fourteen mice were initially randomly separated into two groups: (1) sedentary group (*n* = 7), maintained until they reached 24 months of age, and (2) exercise group (*n* = 7) under a moderate exercise regimen (Monday to Friday; see Table 3, on a treadmill until they reached 24 months of age. A control group (Ctrl) (*n* = 7) of 3-month-old mice was added at the end of the experimental period. All received standard Purina Nutricube food and water ad libitum. After the experimental period, animals were euthanized with sodium pentobarbital, and 100 mg of gastrocnemius tissue was disaggregated with proteinase K for 24 h at 55 °C, treated with dichloromethane, and subjected to liquid–liquid separation to isolate molecules soluble in the dichloromethane fraction; the fraction was air-dried and evaporated.

We also obtained 10 mg of gastrocnemius tissue for dystrophin quantification (MyBiosource, Inc., San Diego, CA, USA, # MBS2023782) in groups and analyzed changes due to aging and exercise.

#### 5.8.4. Analysis of the Presence of 11β-OHP5 in Skeletal Muscle

After extraction, we used an HPLC system to assess 11β-OHP5 in the tissue. We used 11β-OHP5 and pregnenolone as standards. Samples were solubilized in MeOH. Table 4 shows the methodology used. We used 11β-OHP5 and pregnenolone standards and developed concentration curves ranging from 0.01 µg/mL to 125 µg/mL. Extraction recovery of standards was 96–98%. The retention time for 11β-OHP5 in the HPLC analysis was 6.1–6.5 min. The assay sensitivity was between 0.1 and 50 µg/mL, coinciding with the linear part of the concentration curve.

Validation of the range limits was performed according to ICH Q2(R2) guideline; the detection limit (DL) and quantitation limit (QL) were determined based on the standard deviation of the linear response and the slope of a calibration curve according to the formulas: DL = 3.3σ/S and QL = 10σ/S, where σ equals the standard deviation of residuals of the regression line and S equals the slope of the calibration curve. The standard curve was built with seven concentrations ranging from 5 to 25 µg/mL, each in triplicate, and adjusted by ordinary least squares (r^2^ = 0.9927). The QL was verified by an independent analysis of 6 replicates, yielding an RCV of 1.19%. Thus, DL = 2.51 µg/mL and QL = 7.62 µg/mL were obtained.

#### 5.8.5. Statistical Analysis

All data were analyzed using GraphPad Prism 11.0 (GraphPad Software, San Diego, CA, USA). Results are expressed as mean ± standard error of the mean (SEM). We used one-way analysis of variance (ANOVA) with Tukey’s multiple comparisons test for inter-group comparisons, non-linear concentration-response analyses, or t-tests when necessary. Statistical significance was defined as *p* < 0.05, with exact *p*-values reported for all analyses.

## Figures and Tables

**Figure 1:**
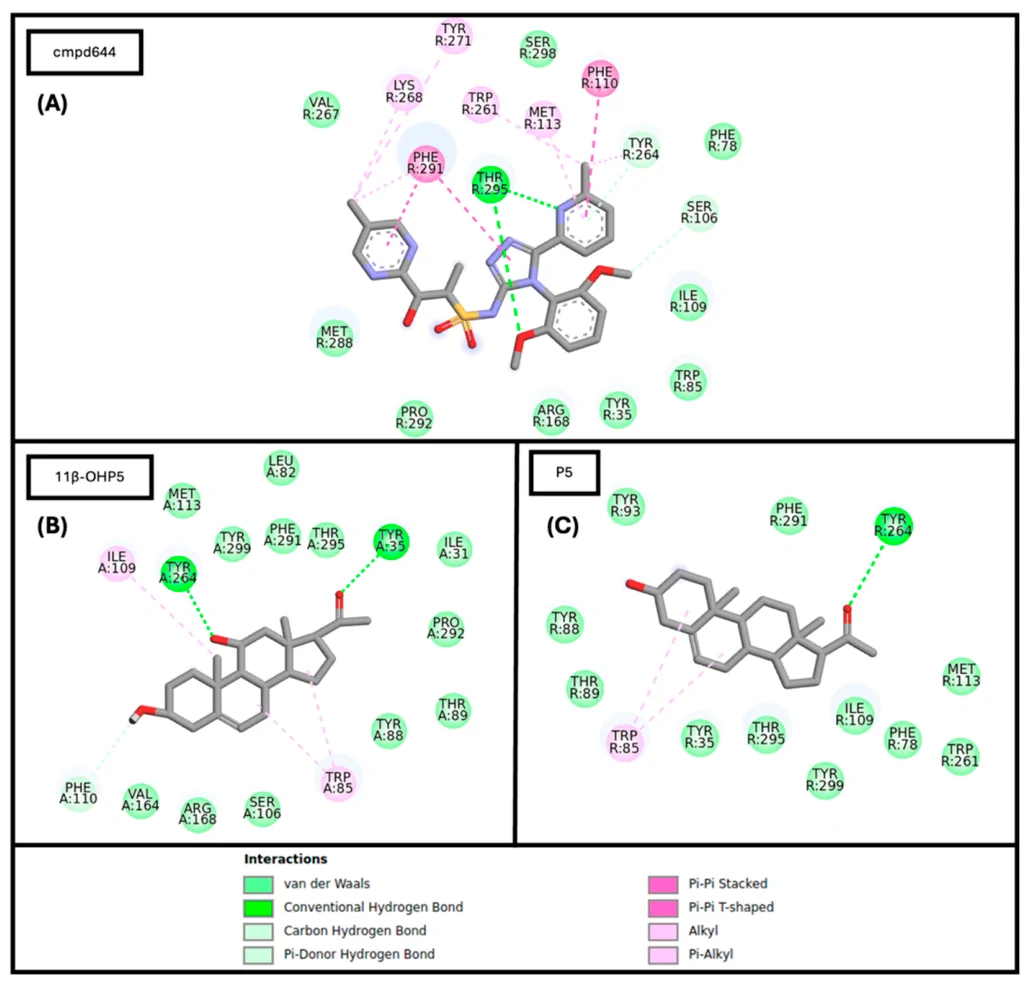
Pose clustering analysis from molecular docking simulations targeting the same binding pocket of aAPLNR. Representative pose clusters from 10,000 independent docking replicates per ligand: (**A**) cmpd644, (**B**) 11β-OHP5, (**C**) P5. Docking simulations were performed with Vina; pose visualization and image generation were performed using Discovery Studio. 11β-OHP5 interacts with Y35, W85, I109, F110, and Y264. Notably, the interactions with F110 and Y264 are shared with cmpd644, while those with W85 and Y264 are shared with P5.

**Figure 2:**
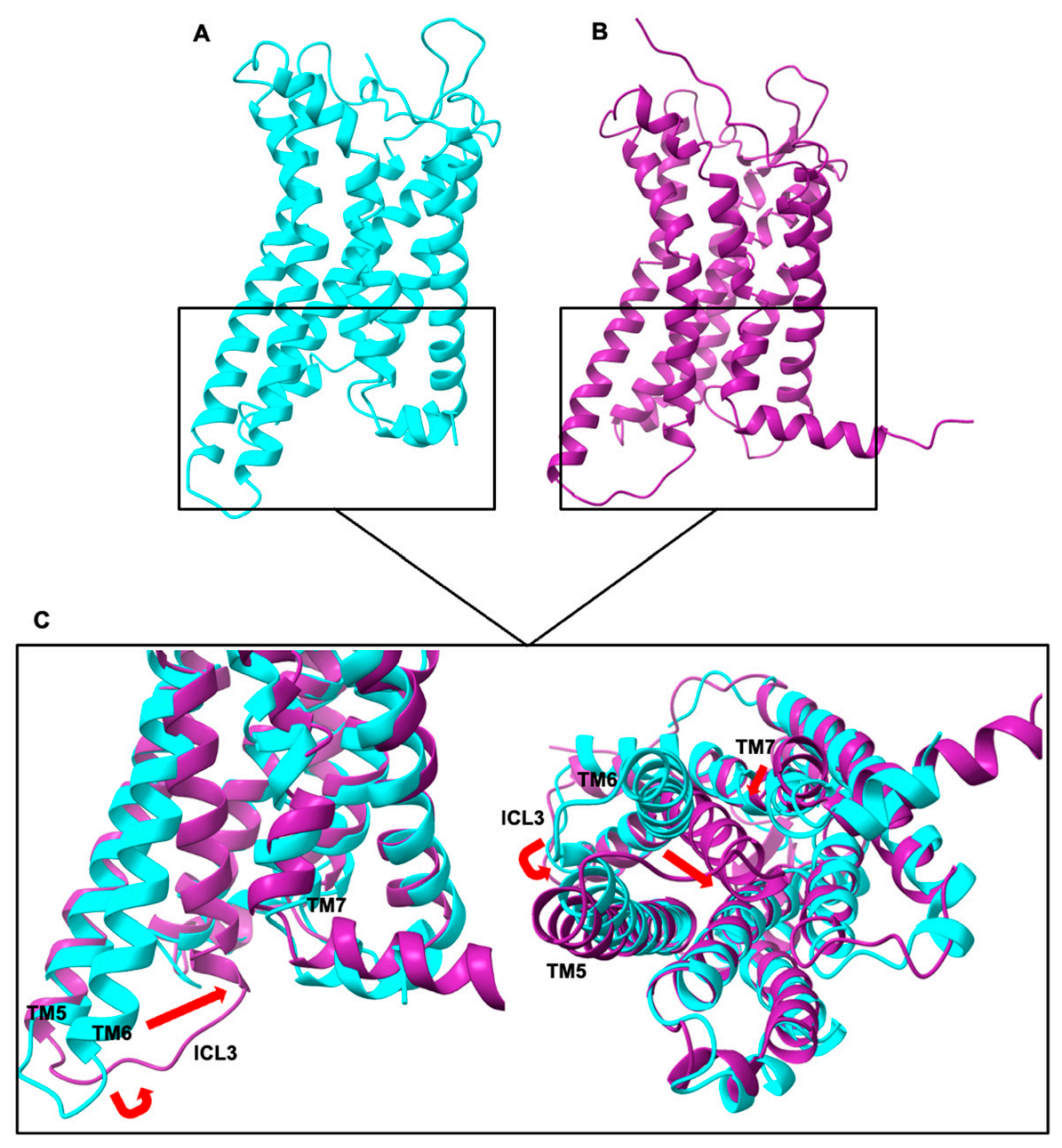
Structural comparison of the crystasllized active and inactive states of APLNR. (**A**) Active state conformation (blue). (**B**) Inactive state conformation (purple). (**C**) Front and bottom views of both superimposed conformations. The transition to the active state is characterized by an outward shift of TM6 away from the receptor core, the adoption of an extended ICL3 conformation perpendicular to the membrane plane, and the approach of TM5 and TM7. These conformational rearrangements expose the critical binding site for effector coupling.

**Figure 3:**
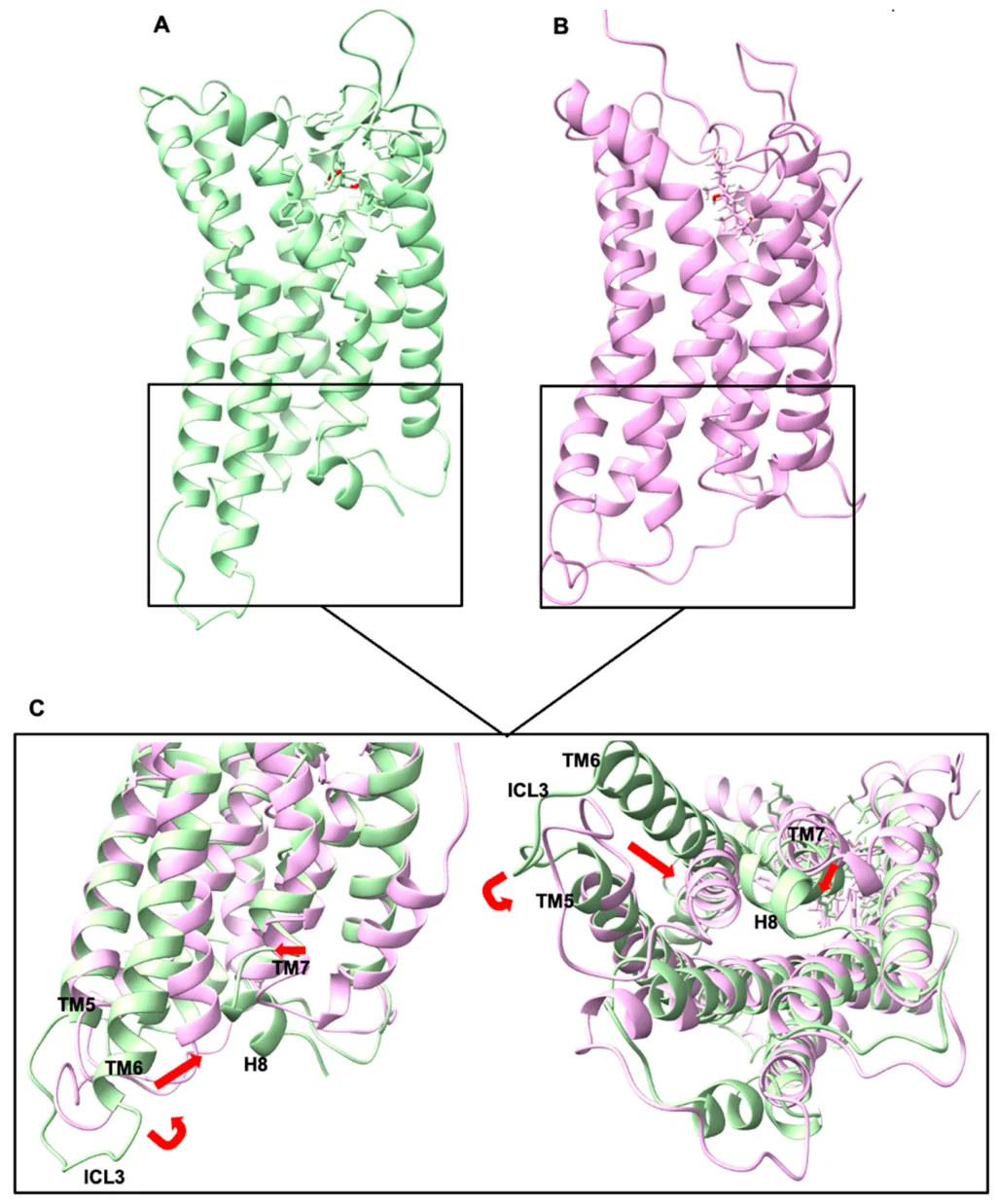
Structural comparison of the active and inactive states of APLNR bound to 11β-OHP5 from molecular dynamics simulations. (**A**) Representative conformation of the majority cluster of the active state (aAPLNR, in green). (**B**) Representative conformation of the majority cluster of the inactive state (iAPLNR, in pink). (**C**) Front and bottom views of both superimposed clusters. The differences found in (**A**) with respect to (**B**), are: (i) TM6 is displaced outward, (ii) ICL3 is in an open form, and (iii) TM7 approaches the receptor core, causing a rearrangement of H8. This collective rearrangement stabilizes a competent binding surface for effector coupling.

**Figure 4:**
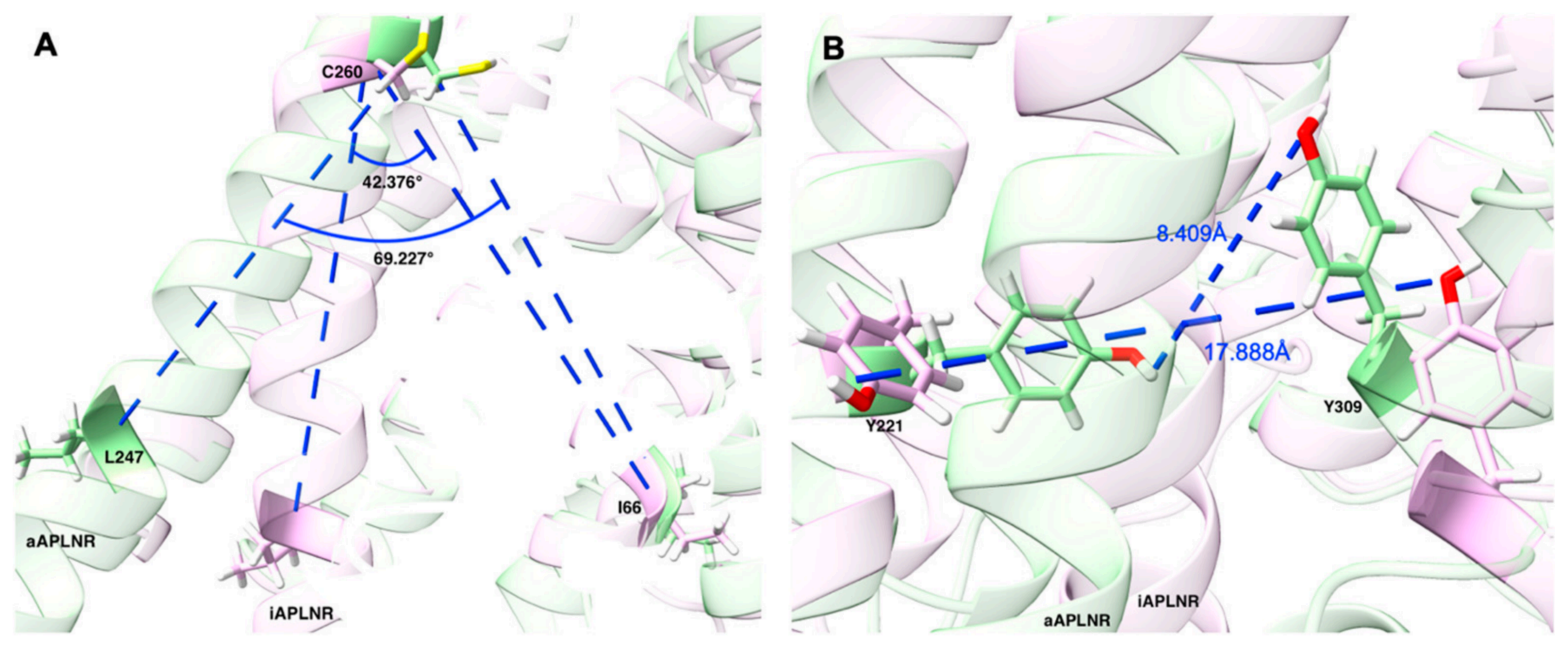
Quantification of conformational changes in the core of APLNR. Geometric analysis of the majority clusters of the active (aAPLNR, green) and inactive (iAPLNR, pink) states. (**A**) Flexion angle of the intracellular segment of TM6, calculated from the alpha carbons of residue L247, C260, and I66. (**B**) Distance between the hydroxyl groups of Y221 (TM5) and Y309 (TM7). The angular distance shown in aAPLNR is characteristic of an active state, while that shown in iAPLNR remains inactive. Similarly, the distance between the tyrosine OH groups decreases. This latter observation suggests stabilization of the active state by narrowing the effector binding zone.

**Figure 5:**
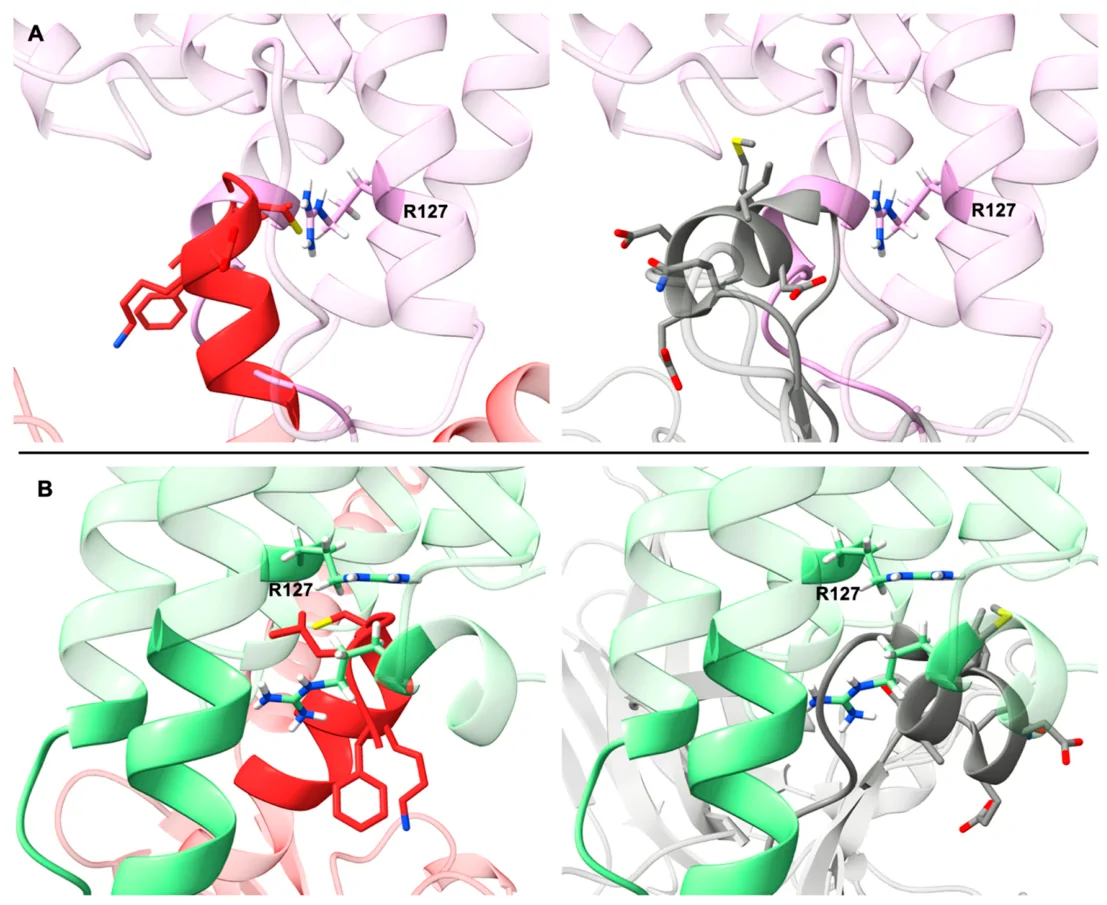
Effector coupling in the active and inactive states of APLNR bound to 11β-OHP5 from molecular dynamics simulations. (**A**) Representative conformation of the majority cluster of the inactive state (iAPLNR, in pink) with the G_i_ protein (Hα5 helix in red) and β-arrestin (finger loop in gray). (**B**) Representative conformation of the majority cluster of the active state (aAPLNR, in green) with the same effectors. R127 is ideally positioned to couple both effectors in both states. The active state releases the steric clashes caused by TM6 and ICL3 with the two effectors. However, the reorientation of the H8 helix in the active state introduces new minor steric clashes.

**Figure 6:**
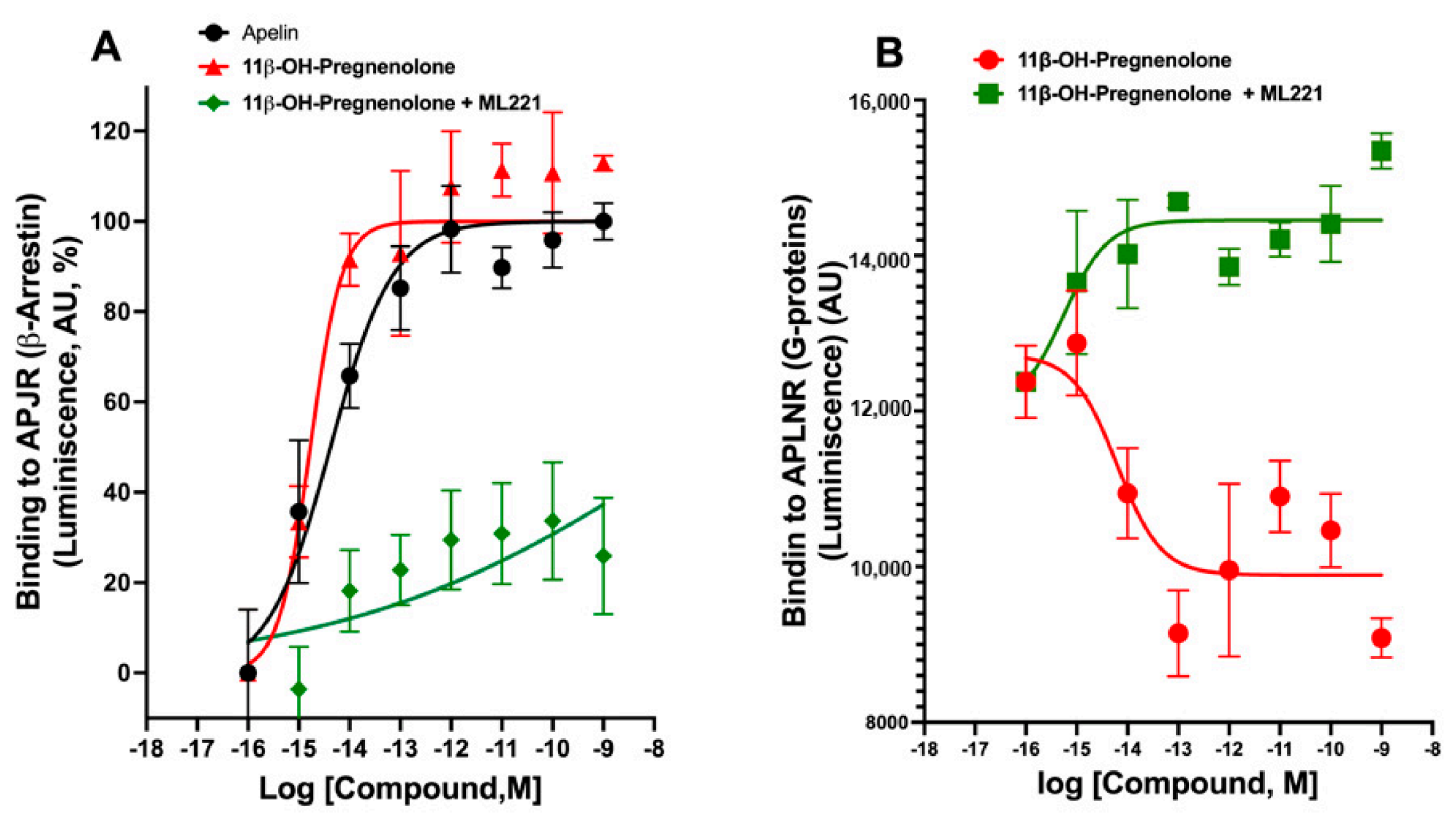
(**A**) Concentration/response of 11β-OHP5 functional activation ofAPLNR. Apelin-13 was used as a positive control. ML221, an APLNR antagonist, was used to assess possible blockage of 11β-OHP5 binding to APLNR. β-arrestin pathways. (**B**) Concentration/response signaling induction of 11β-OHP5 binding to APLNR. Apelin-13 was used as a positive control. ML221, an APLNR antagonist, was used to assess whether 11β-OHP5 binding to APLNR was blocked. GPCR pathway.

**Figure 7:**
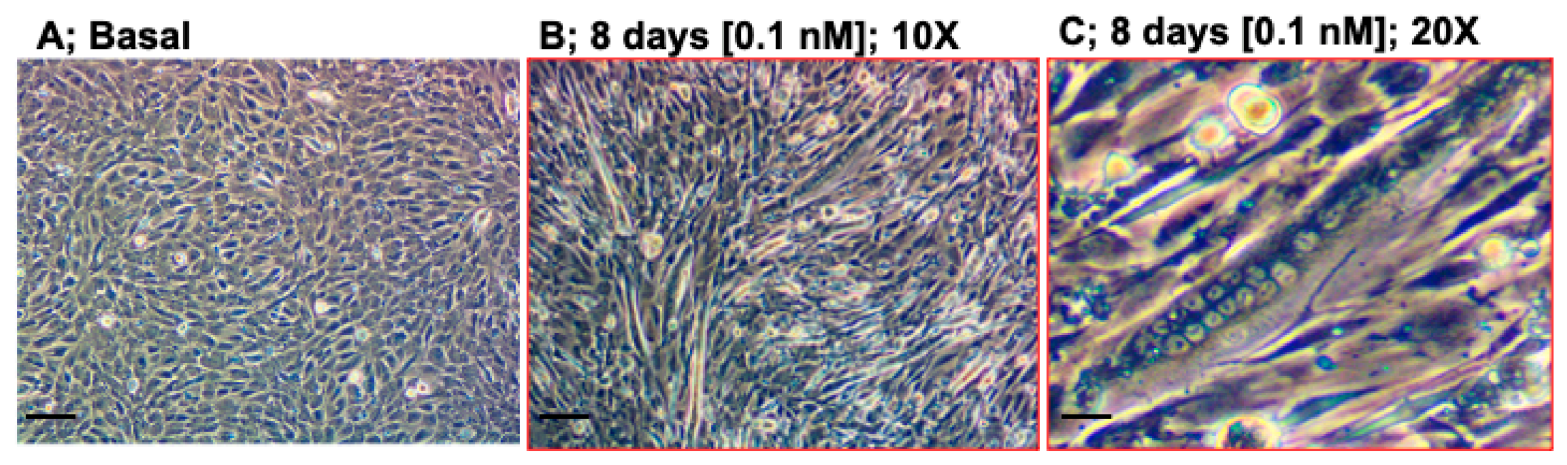
11β-OHP5 enhances myogenic differentiation in C2C12 cells. Microscopic representative images (10× and 20×) of C2C12 cells post-induction of differentiation. Cells were treated with 0.1 nM 11β-OHP5 ((**B**); 10× and (**C**); 20×) or vehicle ((**A**); control). The treated group showed greater differentiation, with myotubes that were more elongated, wider, and contained more nuclei. Bar represents 20 μm in (**A**,**B**) and 40 μm in (**C**).

**Figure 8:**
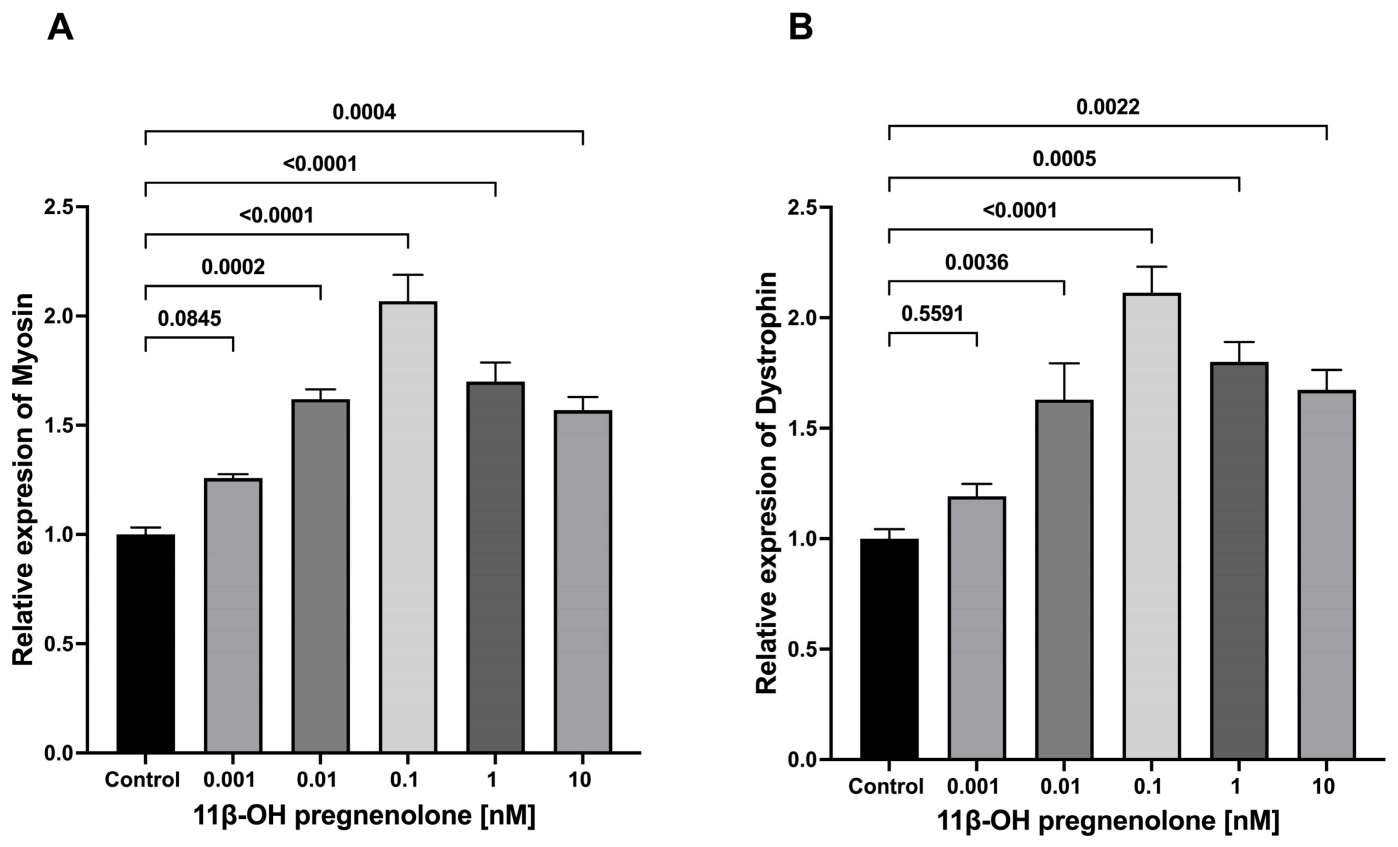
Expression of myogenic differentiation markers in response to 11β-OHP5 treatment. (**A**) *Myosin* (myoblast-to-myotube differentiation marker) mRNA expression. (**B**) *Dystrophin* (myotube-to-myofiber differentiation marker) mRNA expression. Peak expression for both genes occurred at 0.1 nM 11β-OHP5. mRNA expression was quantified by RT-qPCR, normalized to β-actin (reference gene), and expressed as ΔΔCt fold change relative to untreated controls. Data are presented as mean ± SEM (*n* = 3 independent replicates analyzed in triplicate). Exact *p*-values < 0.05 denote statistically significant differences. Statistical analysis was conducted using one-way ANOVA followed by Tukey’s post hoc test.

**Figure 9:**
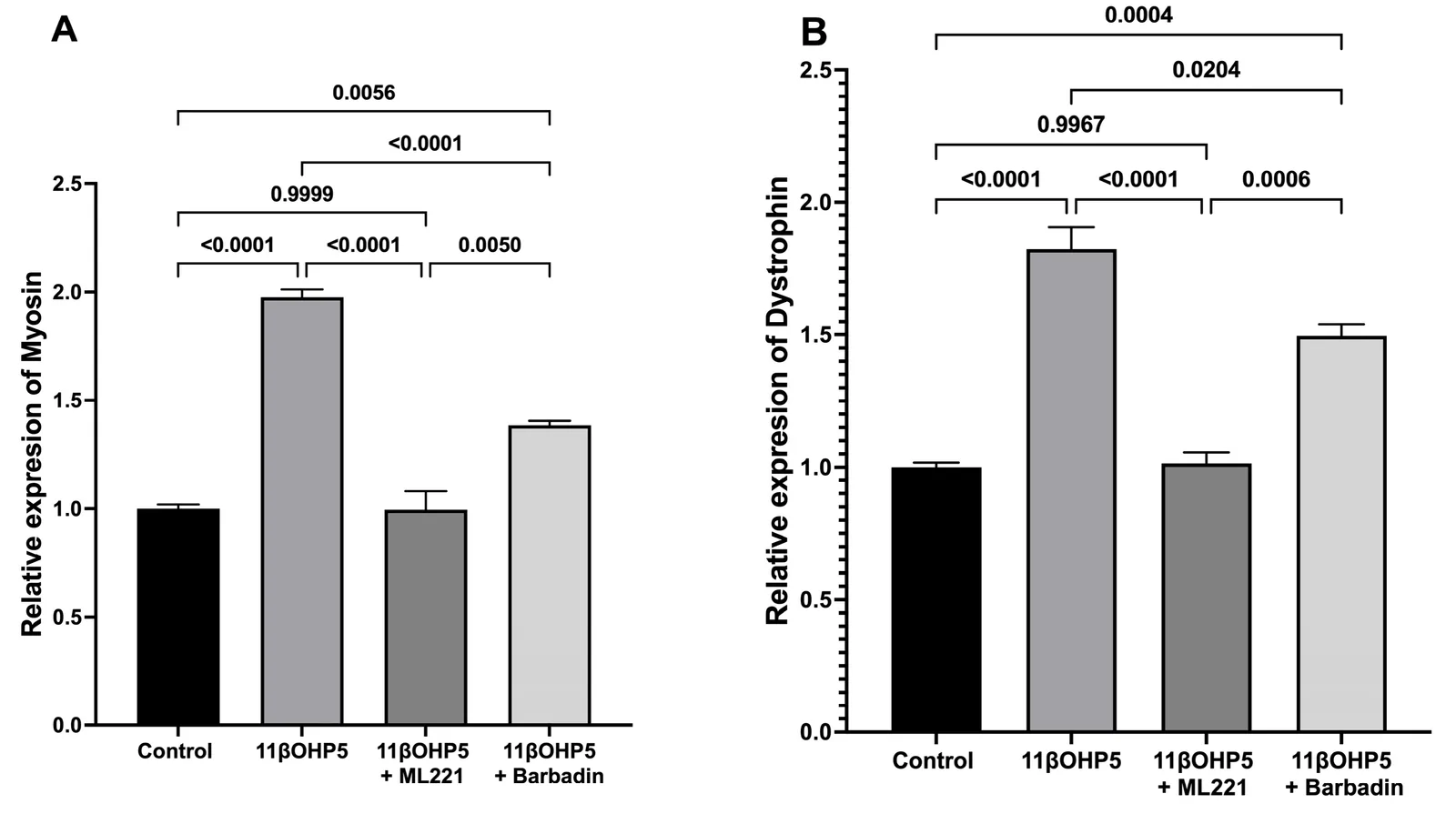
Essential role of APLNR in 11β-OHP5-induced myogenic differentiation. (**A**) Myosin mRNA expression. (**B**) Dystrophin mRNA expression. Complete inhibition by ML221 establishes APLNR as the obligate receptor for 11β-OHP5-mediated differentiation. Differential suppression by barbadin suggests stage-specific β-arrestin and G-protein involvement in myogenesis. mRNA expression was quantified by RT-qPCR, normalized to β-actin, and expressed as ΔΔCt fold change relative to untreated controls. Data are presented as mean ± SEM (*n* = 3 independent replicates). *p* values < 0.05 denote statistically significant differences. Statistical analysis was performed using one-way ANOVA followed by Tukey’s post hoc test.

**Figure 10:**
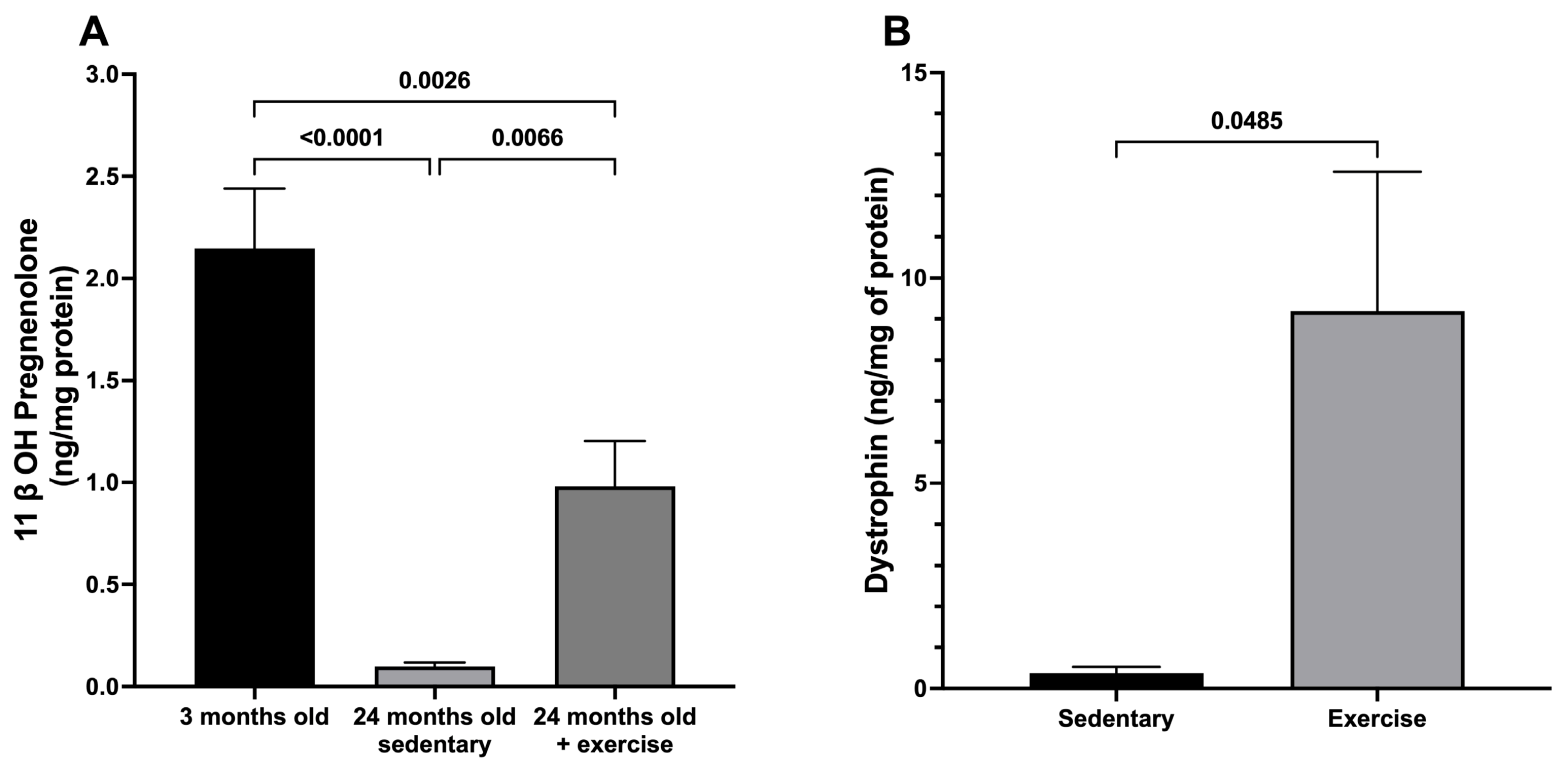
(**A**) Analysis of the concentration of 11β-OHP5 in skeletal muscle of 3-month-old mice and the comparison of concentrations found in sedentary or exercised 24-month-old mice. Data are expressed as mean ± sem (*n* = 7 per group). One-way ANOVA followed by Tukey’s test was used to compare statistical significance. (**B**) Comparison of the concentration of Dystrophin in skeletal muscle found in sedentary or exercised 24-month-old mice. Data are expressed as mean ± sem (*n* = 7 per group). ANOVA was followed by Tukey’s test to compare statistical significance, or a t-test when necessary.

**Table 1:** Energetic decomposition per residue for the binding of 11β-OHP5 to the active and inactive states of APLNR during MD simulations. We calculated free energy of interaction values using the MM/GBSA method. Only residues that contributed significantly to binding throughout the entire MD were included. Values represent the mean ± standard deviation (S.D.). The active complex is more stable than the inactive one.

aAPLNR with 11β-OHP5	Y88					Y93					R168					C181					F291					P292					11β-OHP5	Total	ΔS Aproximation
mean ∆G (kcal/mol)	−0.71					−0.74					−1.11					−0.54					−1.01					−1.33					−14.69	−23.91	−8.29
S.D	0.28					0.49					0.89					0.31					0.53					0.41					2.51	0.56	3.64
**iAPLNR with** **11β-** **OHP5**	**W85**						**I109**						**R168**						**F291**						**T295**						**11β-** **OHP5**	**Total**	**Δ** **S Aproximation**
mean ∆G (kcal/mol)	−0.89						−0.73						−0.94						−0.66						−0.86						−8.05	−12.61	+3.25
S.D	0.6						0.33						0.08						0.42						0.44						2.28	0.87	4.63

**Table 2:** Oligonucleotides used.

Gen	Forward (F)	Reverse (R)
*β-actin*	CATTGCTGACAGGATGCAGA	TGCTGGAAGGTGGACAGTGA
*Myosin*	AGAGCCAAGAGGAAACTGGAGG	CTCGTCCTCAATCTTGCTCTGC
*Dystrophin*	GTGGGAAGAAGTAGAGGACTGTT	AGGTCTAGGAGGCGTTTTCC

**Table 3:** Exercise regimen.

	Velocity	Weekdays
Warmup (1–3 min)	6–10 m/min	Monday–Friday
Exercise (3–25 min)	10–25 m/min	Monday–Friday
Cool off (1–3 min)	25–0 m/min	Monday–Friday

**Table 4:** HPLC methodology.

Column	Eclipse XDB-Phenyl (4.6 × 150 mm) 5 mm particle size	
wavelength	193 and 195 nm + spectral scan (190–400 nm)	
Temperature	25 °C	
Flow	(Quant pump, G1311B): 0.8 mL/min	
Solvents	A = water	B = Acetonitrile
Time (min)	A (%)	B (%)
0	60	40
10.5	35	65
11.5	60	40
15	60	40

## Data Availability

Data are available upon request.

## Supplementary Materials

The following supporting information can be downloaded at: https://www.mdpi.com/article/10.3390/ijms27209379/s1.
