# Supplementary material for: Binding of a Novel 11β-Hydroxysteroid to the Apelin Receptor (APLNR) and Its Possible Role in Regulating Skeletal Muscle Health in Mice
Source: Int J Mol Sci. 2026 Oct 21;27(20):9379. doi: 10.3390/ijms27209379 (PMC13705576; doi:10.3390/ijms27209379)

Table S1. Per-helix backbone RMSD for all seven transmembrane helices in both systems

	**aAPLNR (nm) (mean ± SD)**	**iAPLNR (nm) (mean ± SD)**
TM1	0.26 ± 0.03	3.15 ± 0.78
TM2	0.62 ± 0.16	3.55 ± 0.89
TM3	0.62 ± 0.16	4.05 ± 0.92
TM4	0.62 ± 0.16	4.17 ± 1.10
TM5	0.85 ± 0.28	4.60 ± 0.95
TM6	0.56 ± 0.14	4.92 ± 0.97
TM7	1.12 ± 0.34	3.32 ± 0.69

Figure S1. Solvent-accessible surface area of hydrophobic transmembrane residues

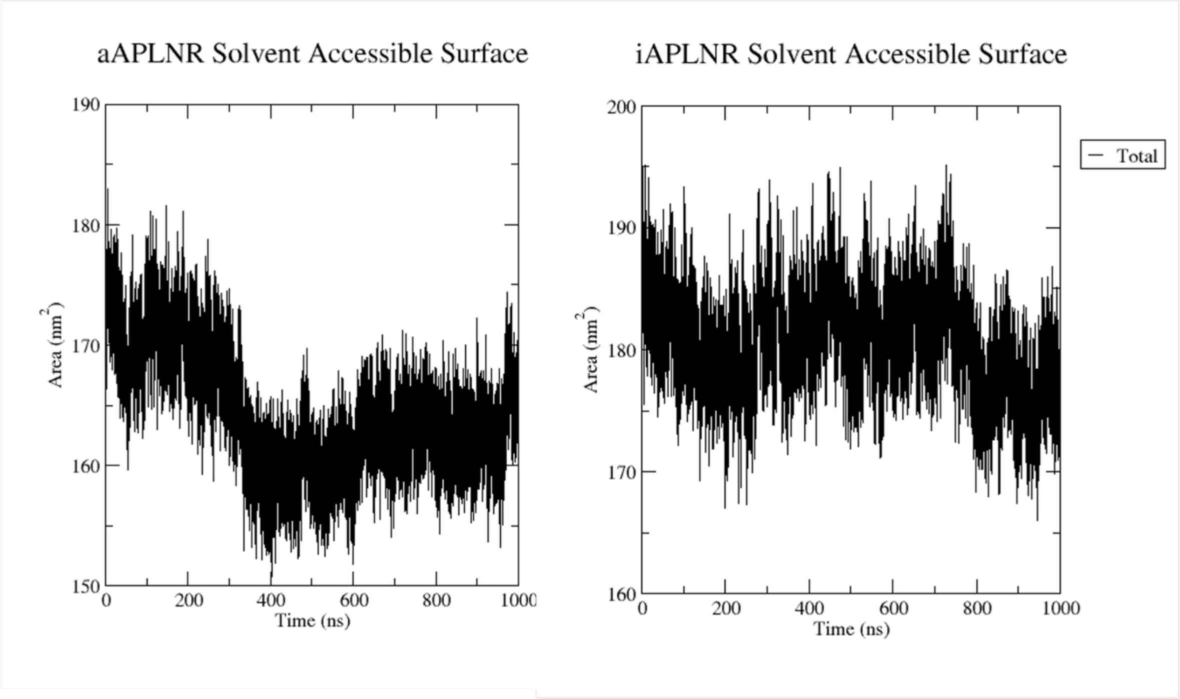

Supplement: Supplementary file 1 [file ijms-27-09379-s001.zip › ijms-4581808-supplementary.pdf]
